# Hemispheric Lateralization of Resting-State Functional Connectivity of the Anterior Insula: Association with Age, Gender, and a Novelty-Seeking Trait

**DOI:** 10.1089/brain.2016.0443

**Published:** 2016-11-01

**Authors:** Sarah Kann, Sheng Zhang, Peter Manza, Hoi-Chung Leung, Chiang-Shan R. Li

**Affiliations:** ^1^ Department of Psychology, State University of New York, Stony Brook, New York.; ^2^ Department of Psychiatry, Yale University School of Medicine, New Haven, Connecticut.; ^3^ Department of Neuroscience, Yale University School of Medicine, New Haven, Connecticut.; ^4^ Interdepartmental Neuroscience Program, Yale University School of Medicine, New Haven, Connecticut.

**Keywords:** aging, hemisphericity, insula, laterality, rsFC, sex difference

## Abstract

Resting-state functional connectivity (rsFC) is widely used to examine cerebral functional organization. The imaging literature has described lateralization of insula activations during cognitive and affective processing. Evidence appears to support a role of the right-hemispheric insula in attentional orientation to salient stimulus, interoception, and physiological arousal, and a role of the left-hemispheric insula in cognitive and affective control, as well as perspective taking. In this study, in a large data set of healthy adults, we examined lateralization of the rsFC of the anterior insula (AI) by computing a laterality index (LI) of connectivity with 54 regions from the Automated Anatomic Labeling atlas. At a corrected threshold (*p* < 0.001), the AI is left lateralized in connectivity with the dorsomedial prefrontal cortex, superior frontal gyrus, inferior frontal cortex, and posterior orbital gyrus and right lateralized in connectivity with the postcentral gyrus, supramarginal gyrus, and superior parietal lobule. In gender differences, women, but not men, showed right-lateralized connectivity to the thalamus. Furthermore, in a subgroup of participants assessed by the tridimensional personality questionnaire, novelty seeking is correlated with the extent of left lateralization of AI connectivity to the pallidum and putamen in men and with the extent of right lateralization of AI connectivity to the parahippocampal gyrus in women. These findings support hemispheric functional differentiation of the AI.

## Introduction

The anterior insula (AI) integrates inputs from cortical and subcortical structures to support emotional and cognitive processes (Craig, 2002, 2009). Functional lateralization of the AI has been observed for interoception in association with the autonomic activity. The right AI increases activation to internal focus on physical and emotional states (Critchley et al., 2004; Napadow et al., 2013; Zaki et al., 2012). In contrast, the left AI is implicated in affiliative behaviors in macaques in response to electrical stimulation (Caruana et al., 2011). Sympathetic and parasympathetic projections from the ventromedial nucleus of the thalamus are lateralized to the right and left AI, respectively (Craig, 2005). These lateralized projections result in differential autonomic control by the AI, such that direct stimulation of the right and left insula, each produces sympathetic and parasympathetic cardiac effects (Oppenheimer et al., 1992). In humans, left AI increases activation to perception of others experiencing an emotion more so than the right AI (Caria et al., 2010; Singer et al., 2004; Wicker et al., 2003), with the level of activation varying with individual rating of empathy (Singer et al., 2004). The left AI also responds both when participants smell a disgusting odor and when they observe others smelling the odor (Wicker et al., 2003), with activity increasing to negative valence ratings of stimuli (Caria et al., 2010). A recent review reported hemispheric dominance of the left insula in emotion perception (Duerden et al., 2013), providing support for cerebral lateralization in affective processing, but also noting inconsistencies due to task designs and highlighting the limitations of exploring functional laterality within a single paradigm.

Other studies support gender differences in emotional processing that involve lateralized insula responses (Duerden et al., 2013; Wager et al., 2003). During exposure to emotional stimuli, the left AI was activated more than the right AI in men, whereas both hemispheres activated to similar levels in women (Duerden et al., 2013). In an earlier meta-analysis, men had greater activation in the left AI, compared to women, in response to emotional stimuli (Wager et al., 2003). However, other studies reported left insula activation to aversive stimuli following negative mood induction by olfaction in women, but not in men (Koch et al., 2007) and covariance of women's, but no men's, subjective rating of a cartoon's funniness with the left insula activity (Kohn et al., 2011). Thus, gender differences in functional lateralization of the insula may be task specific.

The AI shows functional lateralization in cognitive processes. The AI, along with medial prefrontal cortex and subcortical structures, including the thalamus and midbrain, forms the salience network (SN) and responds to infrequent and behaviorally relevant stimuli (Ham et al., 2013; Seeley et al., 2007). Dynamic causal modeling suggests that the right AI is the most likely input to the SN during error processing (Ham et al., 2013). The SN is functionally connected to the default mode network (DMN) as well as the central executive network (CEN). Right AI connectivity modulates DMN activity during cognitive performance (Bonnelle et al., 2012; Sridharan et al., 2008). In patients with traumatic brain injury, the extent of damage to the white matter tracts between the medial prefrontal cortex and right AI predicts less deactivation of the DMN during response inhibition in a stop signal task, with the patient group as a whole showing less DMN deactivation than healthy controls (Bonnelle et al., 2012). Granger causality analyses support a causal role of the right AI in switching between the CEN and DMN in an auditory segmentation task, a visual oddball task, and resting state (Sridharan et al., 2008). Together, these results suggest that the right AI may serve as a “neural hub,” channeling inputs into the SN upon error detection and regulating activity within the CEN and DMN to optimize performance. This line of research supports a right-lateralized attention network (Schotten et al., 2011; Sturm et al., 1999) with the AI playing a central role in the orientation of attention to behaviorally salient targets.

In contrast, the left AI appears to be more involved in top-down behavioral modulation (Ham et al., 2013; Späti et al., 2014). Effective connectivity between the left AI and dorsal anterior cingulate cortex correlates with posterror slowing (Ham et al., 2013), and the left AI activity is significantly higher during self than externally attributed trials in a monetary reward task (Späti et al., 2014). The left, but not right, AI responds to preresponse conflicts (Ullsperger et al., 2010). These findings demonstrate the left AI's role in planning and behavioral adjustment. Thus, on a network level, functional lateralization of the AI embodies right hemispheric dominance in attention (Schotten et al., 2011; Sturm et al., 1999) and left hemispheric dominance in cognitive motor control (Barber et al., 2012; Gotts et al., 2013).

Nonetheless, cognitive and affective functions frequently require both top-down and bottom-up processes and engage the insula bilaterally. For instance, bilateral insula increases activation to prolonged RT following anticipation of a stop signal in the stop signal task (Hu et al., 2015), likely reflecting slower accumulation of sensory information through trial by trial learning (Hu et al., 2014). The bilateral AI responds to emotional interference resolution (Levens and Phelps, 2010), risky decisions during gambling (Xue et al., 2010), and cognitive control (Cole and Schneider, 2007; Dosenbach et al., 2007; Yeung et al., 2006). As with affective responses, functional lateralization of the AI during cognitive processes warrants further investigation.

Functional lateralization of the AI may be elucidated by assessing its resting-state functional connectivity (rsFC) to the whole brain. RsFC characterizes how low-frequency blood oxygenation-level dependent (BOLD) signal fluctuations are coordinated between functionally related regions (Biswal et al., 1995; Dosenbach et al., 2007; Fox and Raichle, 2007). Using rsFC, we have previously characterized whole-brain connectivity and the effects of age and medications on many cortical and subcortical areas (Farr et al., 2014; Li et al., 2014; Manza et al., 2015; Zhang and Li, 2012, 2014; Zhang et al., 2012; Zhang et al., 2015). Other investigators have used rsFC to examine functional architecture of the insula in healthy adults (Cauda et al., 2011), and explore its role in attention-deficit hyperactivity disorder (Tian et al., 2006), as well as depression (Liu et al., 2010) and anxiety (Baur et al., 2013).

In this study, we assessed the rsFC of the AI in 250 healthy adults, focusing on lateralization and the effects of age and gender on the extent of lateralization, on the basis of a literature of age-related changes (Mather, 2012) and gender differences in insula functions. Following previous studies, we computed the Laterality Index (LI) for individual brain regions as defined by the automated anatomical labeling (AAL) atlas (Di et al., 2014; Tzourio-Mazoyer et al., 2002). The insula has also been related to novelty seeking (NS) (Rn Enzi et al., 2009; Song et al., 2015) and harm avoidance (HA) (Ma et al., 2014; Markett et al., 2013). In a subgroup of 57 adults, we explored how personality traits, as evaluated by the tridimensional personality questionnaire (TPQ), are related to lateralized rsFC of the AI (Cloninger, 1987; Sher et al., 1995).

## Materials and Methods

### Data set

Resting-state fMRI scans were pooled from three data sets (Leiden_2180/Leiden_2200, Newark, and Beijing_Zang, *n* = 144) from the 1000 Functional Connectomes Project (Biswal et al., 2010) and our own data (*n* = 106). In selecting the data, we tried to include as many subjects as possible to have more stable findings, as in our earlier work (Zhang and Li, 2014; Zhang et al., 2012). We used only datasets acquired under conditions identical to our own (e.g., similar TR, all under 3T, participants scanned with eye closed). Individual subjects' images were viewed one by one to ensure that the whole brain was covered. A total of 250 healthy subjects' resting-state data (18–49 years of age; 104 men; one scan per participant; duration: 4.5–10 min) were analyzed. Table 1 summarizes these data sets. Men and women did not differ in age (25.4 ± 7.2 vs. 24.0 ± 5.8 years; *p* = 0.096).

**Table 1.** Demographic Information and Imaging Parameters of the Resting-State Functional MRI Data Obtained from the Image Repository for the 1000 Functional Connectomes Project and Our Laboratory

*Dataset*	*Subjects*	*Ages (years)*	*Time points*	*TR (s)*	*Slice acquisition order*
Beijing_Zang	31 M/66 F	18–26	225	2	Interleaved ascending
Leiden_2180	10 M/0 F	20–27	215	2.18	Sequential descending
Leiden_2200	11 M/8 F	18–28	215	2.2	Sequential descending
Newark	9 M/9 F	21–39	135	2	Interleaved ascending
Our own	63 M/43 F	19–49	295	2	Interleaved ascending

M, males; F, females; TR, repetition time.

In a subgroup of 57 individuals (20–47 years of age, 18 men) were assessed with the TPQ-short; (Sher et al., 1995). Derived from the 100-item long form of the TPQ (Cloninger, 1987), the TPQ-Short demonstrated reliability and validity. It consists of 44 yes/no questions, which cover the three dimensions: NS (13 items), HA (22 items), and reward dependence (RD; 9 items). Each personality subscale score was calculated by summing the item scores, reverse scored where necessary. A higher subscore each represents a higher level of NS, HA, and RD. We explored whether these personality traits are related to lateralized AI connectivity to each of the 54 brain regions of the AAL atlas (see below).

### Imaging data processing

Brain imaging data were preprocessed using Statistical Parametric Mapping (SPM 8, Wellcome Department of Imaging Neuroscience, University College London, United Kingdom). Images from the first five TRs at the beginning of each trial were discarded to enable the signal to achieve steady-state equilibrium between RF pulsing and relaxation. Standard image preprocessing was performed. Images of each individual subject were first realigned (motion corrected) and corrected for slice timing. A mean functional image volume was constructed for each subject per run from the realigned image volumes. These mean images were coregistered with the high resolution structural image and then segmented for normalization with affine registration followed by nonlinear transformation (Ashburner and Friston, 1999; Friston et al., 1995).

Additional preprocessing was applied to reduce spurious BOLD variances that are unlikely to reflect the neuronal activity (Fair et al., 2007; Fox and Raichle, 2007; Fox et al., 2005; Rombouts et al., 2003). The sources of spurious variance were removed through linear regression by including the signal from the ventricular system, white matter, and whole brain, in addition to the six parameters obtained by rigid body head motion correction. First-order derivatives of the whole brain, ventricular system, and white matter signals were also included in the regression. Cordes et al. suggested that BOLD fluctuations below a frequency of 0.1 Hz contribute to regionally specific BOLD correlations (Cordes et al., 2001). Thus, we applied a temporal band-pass filter (0.009 Hz <f <0.08 Hz) to the time course to obtain low-frequency fluctuations, as in previous studies (Fox and Raichle, 2007; Fox et al., 2005; Lowe et al., 1998).

### Head motion

As extensively investigated in Van Dijk et al. (2012), micro-head motion (>0.1 mm) is an important source of spurious correlations in rsFC analysis (Van Dijk et al., 2012). Therefore, we applied a “scrubbing” method proposed by Power et al. (2012) and successfully applied in previous studies (Power et al., 2012; Smyser et al., 2010; Tomasi and Volkow, 2014) to remove time points affected by head motions. Briefly, for every time point t, we computed the framewise displacement given by ${ \rm{FD}} ( { \rm{t}} ) = \left\vert { \Delta {{ \rm{d}}_{ \rm{x}}} ( { \rm{t}} ) } \right\vert + \left\vert { \Delta {{ \rm{d}}_{ \rm{y}}} ( { \rm{t}} ) } \right\vert +  \left\vert { \Delta {{ \rm{d}}_{ \rm{z}}} ( { \rm{t}} ) } \right\vert + { \rm{r}} \left\vert {{ { \alpha }} ( { \rm{t}} ) } \right\vert + { \rm{r}} \left\vert {{ { \beta }} ( { \rm{t}} ) } \right\vert + { \rm{r}} \left\vert {{ { \gamma }} ( { \rm{t}} ) } \right\vert$, where $( {{ \rm{d}}_{ \rm{x}}},{{ \rm{d}}_{ \rm{y}}},{{ \rm{d}}_{ \rm{z}}} )$ and $( { \alpha , \beta , \gamma } )$ are the translational and rotational movements, respectively, and *r* (=50 mm) is a constant that approximates the mean distance between center of MNI space and the cortex and transforms rotations into displacements (Powers et al., 2012). The second head movement metric was the root mean square variance (DVARS) of the differences in% BOLD intensity I(t) between consecutive time points across brain voxels, computed as follows: ${\rm{DVARS}} ( { \rm{t}} ) = \sqrt {\langle {{ \left\vert {{ \rm{I}} ( { \rm{t}} ) - { \rm{I}} ( {{ \rm{t}} - 1} ) } \right\vert }^2} \rangle }$, where the brackets indicate the mean across brain voxels. Finally, to compute each subject's correlation map, we removed every time point that exceeded the head motion limit FD(t) >0.5 mm or DVARS(t) >0.5% (Power et al., 2012; Tomasi and Volkow, 2012). On average, 1% of the time points were removed across subjects.

### Seed based correlation and group analyses

**FIG. 1.**
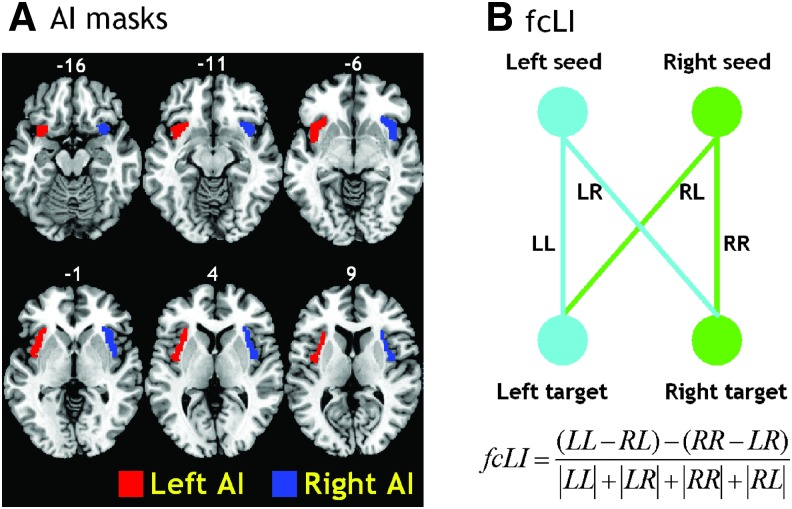
**(A)** Masks of the right and left anterior insula; **(B)** Laterality Index (LI). The value of LI ranges from −1 (R lateralization) to +1 (L lateralization), with a larger absolute value indicating greater lateralization in the connectivity between the seed and target. Color images available online at www.liebertpub.com/brain

The left and right AI masks were generated using both cytoarchitectonic and topographical criteria based on Neuromorphometrics labels (www.neuromorphometrics.com) included in the SPM 12 software package (Fig. 1A). The BOLD time courses were averaged spatially each over the left and right AI seed. For individual subjects, we computed the correlation coefficient between the averaged time course of each seed region and the time courses of all other brain voxels. To assess and compare the rsFC, we converted these image maps, which were not normally distributed, to z-score maps by Fisher's z-transform (Berry and Mielke, 2000; Jenkins and Watts, 1968). The Z maps were used in group random-effect analyses. We performed one-sample t-test each on the Z maps of left and right AI and paired-sample t test comparing the two Z maps.

### Functional connectivity laterality index

A few considerations distinguished the computation of functional connectivity laterality index (fcLI) from the LI employed conventionally to characterize lateralization of cerebral activations to cognitive challenges: (L−R)/(L+R). First, negative connectivity of a brain region to the L (or R) seed cannot be distinguished from positive connectivity to the R (or L) seed. Second, target regions in the same hemisphere of the seed region will always have stronger functional connectivity than their counterparts in the other hemisphere (please see Results below). To manage these issues, therefore, we followed previous studies (Liu et al., 2009) to compute the fcLI based on connectivities of paired seed and target regions between the hemispheres. Briefly, the fcLI was computed as follows:

\begin{align*}
fcLI = { \frac { ( { LL - RL } ) - ( { RR - LR } ) }  { \left\vert { LL } \right\vert + \left\vert { LR } \right\vert + \left\vert { RR } \right\vert + \left\vert { RL } \right\vert } } 
\end{align*}

where *LL* is the functional connectivity between the *L* seed and *L* target region; *RR* is the functional connectivity between the *R* seed and *R* target region; *RL* is the functional connectivity between the *R* seed and *L* target region; and *LR* is the functional connectivity between the *L* seed and *R* target region (Fig. 1B). As computed, positive fcLI indicates left lateralization; that is, the target region, irrespective of its hemisphericity, has more connectivity to the *L* than *R* seed region. By contrast, a negative fcLI indicates right lateralization. The value of fcLI ranges from −1 (*R* lateralization) to +1 (*L* lateralization), with a larger value indicating greater lateralization in the connectivity between the seed and target.

In the sample assessed for TPQ, we examined whether NS and HA traits are related to lateralized AI connectivity to each of the 54 brain regions of the AAL atlas. Because of multiple tests, an alpha of 0.05/(54 × 2)–0.0005 would be required to guard against Type I error. However, we considered that not all of the 54 brain regions should be considered independent from one another, and used a *p* < 0.001 to examine the pair-wise regressions.

## Results

### Differences in whole-brain connectivity between right and left insula

**FIG. 2.**
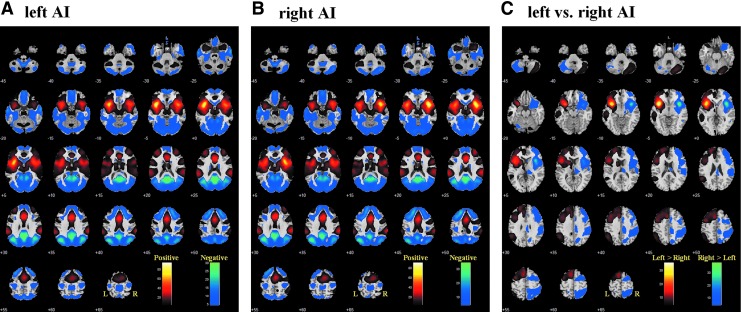
**(A)** Whole-brain functional connectivity of the **(A)** left and **(B)** right AI; **(C)** differences in whole-brain functional connectivity of the left versus right AI. *p* < 0.05, FWE corrected. AI, anterior insula.

Figure 2A, B each shows the whole-bran rsFC of the left and right AI at *p* < 0.05 corrected for family-wise error of multiple comparisons. A direct contrast between these two maps demonstrated the left and right AI each with greater connectivity (greater positive or less negative connectivity) to regions in the same hemisphere (Fig. 2C).

### Lateralized regional connectivities to the AI

To examine whether the AI shows lateralized cerebral connectivity, one needs to go beyond this intrinsic, “biased” pattern of connectivity. An important question to ask is whether a given brain region is more connected to the left or right AI irrespective of the regions' hemisphericity. To this end, we followed previous studies (Liu et al., 2009) to derive a lateralization index (LI) of connectivity between each of the 54 brain regions with both L and R hemispheric masks from the AAL atlas.

**FIG. 3.**
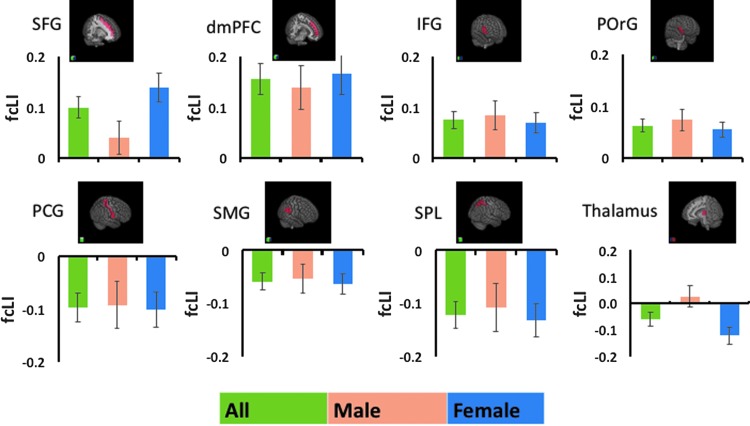
Brain regions with a significant LI of functional connectivity (fcLI) to the AI. Bar plots show mean ± standard error of fcLI for men and women combined (green), and men (orange) and women (blue) separately. The top row shows regions with positive LI or left lateralization of AI connectivity. The bottom row shows regions with negative LI or right lateralization of AI connectivity.

**FIG. 4.**
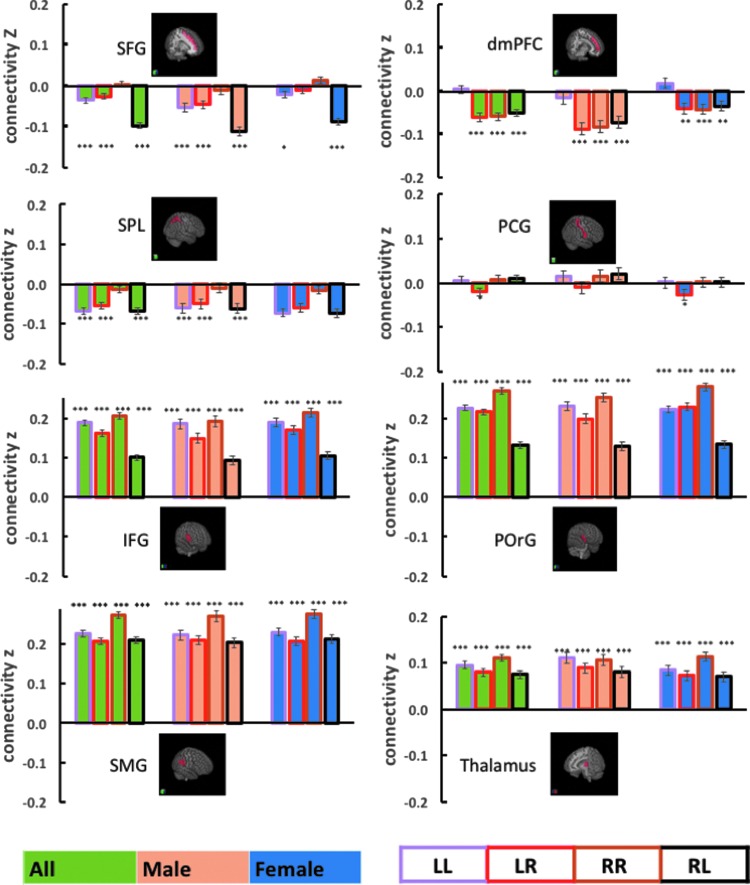
Mean ± standard error for the connectivity z value for the eight target regions with lateralization in AI connectivity, broken down according to gender (men vs. women), AI (L vs. R), and target region (L vs. R) hemisphericity. Significance of the connectivity was further examined by one-sample t-test against zero for z values and marked with ***p < 0.001, **p < 0.01, and *p < 0.05.

The results showed that, at a corrected threshold (*p* < 0.05/54–0.001), the superior frontal gyrus (SFG), inferior frontal gyrus (IFG), posterior orbital gyrus (POrG), and dorsal medial prefrontal cortex (dmPFC) showed left lateralization. The postcentral gyrus (PCG), superior parietal lobule (SPL), and supramarginal gyrus (SMG) showed right lateralization (Fig. 3). A two-sample *t*-test showed that the left lateralization of SFG connectivity is significantly greater in women than in men (*p* < 0.019). Of note, although the thalamus did not show significant lateralization within the general group analysis, women showed a significant right lateralization (*p* < 0.001), whereas men did not (*p* > 0.050). This gender difference was confirmed in a two-sample *t*-test of the LI (*p* < 0.005). In Figure 4, we showed the connectivity measures broken down for L and R seed and target regions.

### The effect of age on lateralized functional connectivity of the AI

We assessed whether age influences lateralization of the rsFC of the AI. The extent of left lateralization of AI connectivity to the SFG was negatively correlated with age in men (*r* = −0.33, *p* < 0.001), but not in women (*r* = 0.03, *p* = 0.67), while the correlation for the group as a whole was not significant at a corrected threshold (*r* = −0.15, *p* = 0.02). Men and women showed a significant difference in slope of the linear regressions (*p* = 0.001). It should be noted that the SFG showed significantly left lateralization for women, but not for men, with a significant gender difference (see above). It thus appears that as men age, AI connectivity to the SFG becomes significantly less left lateralized, while women continue to maintain left lateralization in connectivity to the AI.

### The effect of an NS trait on lateralized functional connectivity of the AI

**FIG. 5.**
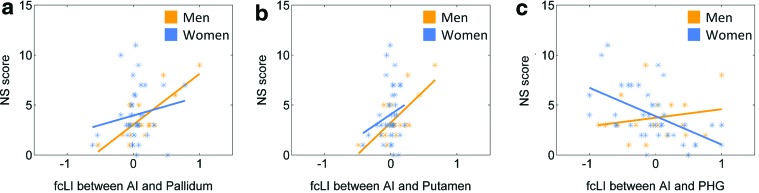
Linear regression of the LI of **(A)** pallidum, **(B)** putamen, and **(C)** parahippocampal gyrus against novelty seeking (NS) subscore each for men (orange) and women (blue). Color images available online at www.liebertpub.com/brain

In a smaller cohort of subjects evaluated with the TPQ, we examined whether HA, RD, and NS subscore correlate with the LI at a corrected threshold (*p* < 0.001/3–0.00033). The results showed that NS subscore is positively correlated with the LI of the pallidum in men (*p* < 0.0002, *r* = 0.76), but not in women (*p* = 0.3134, *r* = 0.17). At a less stringent threshold of *p* < 0.001, NS subscore is also positively correlated with the LI of the putamen in men (*p* < 0.0009, *r* = 0.71), but not in women (*p* = 0.2007, *r* = − 0.21), and negatively correlated with the LI of the parahippocampal gyrus (PHG) in women (*p* < 0.0006, *r* = −0.53), but not in men (*p* = 0.4329, *r* = 0.20). That is, NS is associated with left lateralization of AI connectivity to the pallidum and putamen in men and with right lateralization of AI connectivity to the PHG in women. Figure 5 shows scatter plots and linear regressions of these correlations. However, a test of slope difference in regression between men and women was significant for the PHG (*p* < 0.01), but not the palladium or putamen (*p'*s > 0.05). Of note, men and women did not differ in age (28.1 ± 7.2 vs. 26.1 ± 5.3 years; *p* = 0.23) or in NS subscore (3.7 ± 2.2 vs. 4.0 ± 2.7; *p* = 0.68) in this cohort.

## Discussion

### rsFC of the AI

Our findings broadly corroborated those of previous studies that assessed rsFC of the insula, with the anterior network connected with the medial prefrontal regions (including the anterior cingulate cortex), middle and inferior frontal gyri, as well as the SMG (Cauda et al., 2011). The discussion below focused on lateralization of functional connectivity of the AI.

### Hemispheric lateralization of the AI rsFC

The AI is right lateralized in connectivity to the PCG, SMG, and SPL. These findings are consistent with the correlated right-hemispheric dorsal AI and SPL/SMG activity in relation to attention performance (Touroutoglou et al., 2012). As part of the ventral attention system, the SMG responds to both auditory (Opitz et al., 1999) and visual (Ardekani et al., 2002) “odd-ball” stimuli. Critical for sensorimotor integration, the SPL responds to attention to a moving stimulus (Molenberghs et al., 2007; Vandenberghe et al., 2001), and SPL lesions compromise visuospatial attention in humans (Vandenberghe et al., 2012). The PCG includes the somatosensory cortex and differentiates salient from frequent sensory inputs in conjunction with the insula (Chen et al., 2010). Neuronal recordings from the primary somatosensory cortex in monkeys related the synchrony of neuronal firings to modulations in attention and task switching (Steinmetz et al., 2000). Human neuroimaging linked prestimulus alpha oscillations and mu rhythm in the somatosensory cortex to attentional demands (Anderson and Ding, 2011; Haegens et al., 2012). Thus, right lateralization of functional connectivity of the AI to these structures supports responses to salient stimuli and attention reorientation for actions.

The dorsomedial prefrontal cortex (dmPFC) and inferior frontal cortex (IFC) were significantly left lateralized in AI connectivity in both women and men. The mPFC includes multiple regions that have been implicated in proactive control (Hu et al., 2015) and error monitoring (Gehring and Willoughby, 2002; Ide et al., 2013; Ridderinkhof and Ullsperger, 2004; Zhang et al., 2012). Likewise, the IFC has shown distinct roles in attention orientation and response inhibition (Cai et al., 2014; Chao et al., 2009; Duann et al., 2009; Leung and Cai, 2007; Swick et al., 2008). Left lateralized connectivity to dmPFC and IFC accords with previous findings linking the left AI to “moment to moment adjustments in behavioral control” (Ham et al., 2013).

The dmPFC also plays a critical role in self-referencing and perspective taking (Northoff and Bermpohl, 2004; Northoff et al., 2006). Positron emission tomography (PET) imaging showed a higher activity in the dmPFC in medical students answering questions from the perspective of a lay person versus a medical professional (Ruby and Decety, 2003). The dmPFC increased activation in participants rating how pleased a person is to have their photo taken, compared to rating the symmetry of facial features (Mitchell et al., 2005). A meta-analysis of 65 imaging studies of emotion found greater activity in the mPFC in approach compared to withdrawal emotions (Wager et al., 2003). Likewise, the IFG and POrG have each been implicated in processing others' emotions (Schulte-Rüther et al., 2007; Wrase et al., 2003) and perspective taking (Hodzic et al., 2009). Also, in support of left lateralized connectivity of the AI to these regions, are previous findings of the left insula responding to perception of others' emotional state (Caria et al., 2010; Singer et al., 2004; Wicker et al., 2003). Taken together, evidence accumulates to support the left AI in empathetic affiliative behaviors (Craig, 2005), which involves an externalized awareness of the self in relation to the other, in concert with parasympathetic autonomic control (Caruana et al., 2011).

### Age-related changes and gender differences in functional lateralization of the AI

The SFG significantly decreased in left-lateralized connectivity to the AI in men as a function of age, in contrast to women who maintained significant left lateralization in rsFC. The SFG is part of the dorsal lateral prefrontal cortex (DLPFC), a functional region implicated in age-related cognitive changes (Harlé Katia and Sanfey, 2012; Langner et al., 2015; MacPherson et al., 2002; Zhu et al., 2014). Compared to young adults, older adults show impaired performance in resolving stimulus–response conflict and decreased connectivity of bilateral AI to dorsomedial PFC and DLPFC (Langner et al., 2015). Older adults, compared to younger adults, showed higher activation in the right DLPFC and right AI within a task-switching paradigm (Zhu et al., 2014). Within unfair offer trials of an economic decision task, older adults exhibited higher activation in the DLPFC and lower activation in bilateral AI relative to younger adults (Harlé Katia. and Sanfey, 2012). Last, a study found a positive relationship between a measure of BOLD variability (SD_BOLD_) of the SFG and insula during resting state and performance on a memory task in older adults (Burzynska et al., 2015). These findings, however, do not address why the age-related changes transpire in men, but not in women.

Thalamic connectivity to the AI was significantly right lateralized in women, but not in men, with the LI being significantly different between genders. The insula receives input from the ventromedial thalamus (Barbaresi et al., 1992.; Cechetto and Saper, 1987; Friedman et al., 1986), a region that encodes nociceptive stimulus intensity (Hutchison et al., 1994). Women had a significantly higher activity, as measured by PET imaging, in the AI and thalamus when experiencing heat stimuli and rated heated stimuli as more painful than men (Paulson et al., 1998). In studies of micturition control, the right AI and midbrain periaqueductal gray were more active at higher than at lower bladder volumes, and responses of the right thalamus and several other right hemispherical regions were stronger in women than in men (Kuhtz-Buschbeck et al., 2009). Along with these studies, the finding of right-lateralized AI thalamic connectivity supports higher sensitivity to salient somatosensory and interoceptive stimuli in women. Multiple studies have demonstrated gender differences in behavior on tasks involving both pain (for review see Fillingim and Maixner, 1995) and emotional sensitivity (Dimberg and Lundquist, 1990; Kring and Gordon, 1998), with insula responding differently between men and women in various emotional tasks (for review see Duerden et al., 2013). Right-lateralized connectivity between the AI and thalamus in women, but not men, appears to support gender differences in pain and affective processing.

### NS and functional lateralization of the AI

The personality trait NS describes a tendency to react to novel stimuli intensely and has been associated with reward-seeking behavior, including substance misuse (Cloninger, 1987; Cotto et al., 2010; Sher et al., 1995). Within our sample, men, but not women, showed a positive relationship between NS and left-lateralized connectivity of the AI to the pallidum and putamen. The striatum and insula are heavily connected anatomically (Chikama et al., 1997) and both structures are associated with reward predictions (Tachibana and Hikosaka, 2012; Tanaka et al., 2004; Wittmann et al., 2011). An earlier work linked NS to differential activities of these regions, with novelty response of the left striatum correlated with individual NS score (Wittmann et al., 2008). In another study, NS scores correlated negatively with activity during risk prediction in the left AI and right striatum (Wang et al., 2015). Broadly consistent with these earlier studies, this finding may be further explored for a link to gender differences in neural mechanisms of substance misuse and other addictive behaviors (Cohen et al., 1993; Cotto et al., 2010; Kampov-Polevoy et al., 2004).

The human hippocampal formation is involved in processing novelty signals (Daselaar et al., 2006; Köhler et al., 2005; Kumaran and Maguire, 2009; Schroeder et al., 2004). Unlike the hippocampus, which responds to changes in the relationship between objects and background, the PHG is engaged only by scene novelty, in participants performing an incidental target-detection task (Howard et al., 2011). Our finding of right-lateralized connectivity of AI to PHG supports a mechanism of concerted attention orientation in women. How functional connectivity between the AI and PHG subserves novelty detection remains to be investigated.

## Conclusion

This study assessed lateralized functional connectivity between the AI and cortical and subcortical areas. The results showed a distinct pattern of right lateralization with regions implicated in attention orientation and arousal and left lateralization with regions implicated in cognitive motor control and perspective taking. The pattern of functional lateralization appears to accord with the role of the right and left AI each in sympathetic and parasympathetic autonomic responses (Craig, 2005) as well as hemispheric lateralization of neural networks to support bottom-up and top-down processing (Gotts et al., 2013). Our findings also suggest that functional lateralization of the AI may vary with age, gender, and personality traits.

A few limitations need to be considered. First, we could not study the functional implications of the patterns of lateralized connectivity because participants were not assessed for neurocognitive performance. Second, although we reported age-related effects, this sample included only young and middle-aged adults and the findings should be considered specific to this age range. Third, in discussing these findings, we sometimes referred to an imaging literature that does not always distinguish between the anterior and posterior insula. These important issues need to be addressed in future work.
